# A Rare Lesion in the Anterior Region of the Third Ventricle: Rosette-Forming Glioneuronal Tumor

**DOI:** 10.7759/cureus.51971

**Published:** 2024-01-09

**Authors:** Leopoldina Pereira, Djamel Kitumba, Mário Gil Fontoura, David A João, Lino Mascarenhas, Mário Resende

**Affiliations:** 1 Neurosurgery, Centro Hospitalar Vila Nova de Gaia/Espinho, Vila Nova de Gaia, PRT; 2 Neurosurgery, Complexo Hospitalar de Doenças Cardio-Pulmonar Cardial Dom Alexandre do Nascimento, Luanda, AGO; 3 Internal Medicine, Centro Hospitalar de Entre Douro e Vouga, Santa Maria da Feira, PRT; 4 Pathology, Centro Hospitalar Vila Nova de Gaia/Espinho, Vila Nova de Gaia, PRT; 5 Neurological Surgery, Centro Hospitalar Vila Nova de Gaia/Espinho, Vila Nova de Gaia, PRT

**Keywords:** seizures, endoscopy, cerebral salt-wasting syndrome, third ventricle, rosette-forming glioneuronal tumor

## Abstract

Rosette-forming glioneuronal tumor (RGNT) is a rare and indolent mixed glioneuronal tumor involving primarily the fourth ventricular region and occurring predominantly in young adults.

We present a case of a 44-year-old woman presented with progressive headaches, vomiting, and a sudden decreasing level of consciousness. The magnetic resonance imaging showed a regular lesion within the anterior portion of the third ventricle and the patient underwent an endoscopic approach to remove the tumor that was exclusively within the anterior portion of the third ventricle. Histopathology showed an RGNT that was totally removed.

We also report some unusual complications that are described in the literature and are related to ventricular endoscopy such as seizures and hydroelectrolyte disorders.

With two years of follow-up, the patient had no complaints and no tumor progression was observed.

## Introduction

Rosette-forming glioneuronal tumor (RGNT) is a rare and indolent brain tumor and it was originally thought to be exclusively localized into the fourth ventricle [1]. Nowadays, RGNT has been reported in various brain locations such as the cerebellar hemisphere, vermis, pineal region, optic chiasma, lateral and third ventricle, hypothalamus/thalamus, and also in the spinal cord. However, its location in the anterior portion of the third ventricle with no dissemination into the thalamus or hypothalamus, to the best of our knowledge, has never been reported before [2].

We report a case of an RGNT exclusively from the anterior portion of the third ventricle that occurred in a 44-year-old woman which was managed by a straightforward endoscopic approach and we present some uncommon complications after the surgical approach such as tonic-clonic seizures and cerebral salt wasting syndrome (CSWS). We also carry out an extensive review of the literature about all cases of RGNT on the third ventricle.

## Case presentation

**Figure 1:**
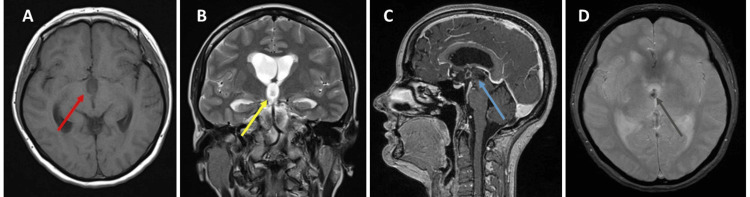
Preoperative MRI - Hypointense in T1 (red arrow) and hyperintense in T2 (yellow arrow) (A and B); T1 with contrast showed a mild dotted enhancement (blue arrow) in the center of the lesion (C); Hypointense signal on the gradient-echo (T2*) (black arrow - D).

 A 44-year-old woman presented in the emergency room (ER) with one week of progressive headaches, vomiting, and a sudden decreasing level of consciousness. On examination, she was drowsy but rousable, Glasgow Coma Scale (GCS) score of 14/15. Her cranial nerves were all intact with no diplopia and the exam showed normal strength (5/5) - by the Medical Research Council motor scale - normal sensation, reflexes, and coordination in both upper and lower extremities. The magnetic resonance imaging (MRI) of the head showed a well-defined lesion of 26.6x18.2x10.7mm with irregular borders within the anterior portion of the third ventricle. It was hypointense on T1 and hyperintense on T2 sequences. Contrast enhancement showed mild dotted enhancement inside the mass with a hypointense signal on the gradient-echo (T2*) (Figure 1).

The sagittal image showed that the lesion only involved the anterior portion of the third ventricle, obstructing the foramen of Monro, with no invasion of the third ventricle recesses. Coronal image showed ventriculomegaly only of the lateral ventricles with no invasion of the lesion to the lateral walls of the third ventricle. No satellite lesion was noted and there were no abnormal findings in other regions of the brain.

**Figure 2:**
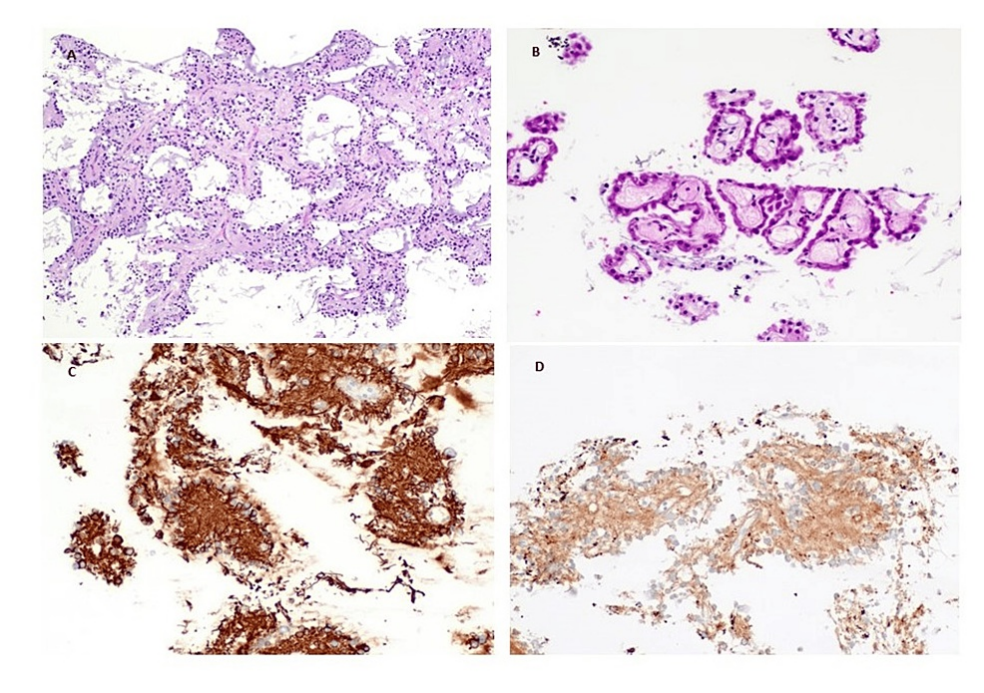
Histopathological findings of brain biopsy. On hematoxylin and eosin staining, fragments of a biphasic, moderately cellular neoplasm were found, comprised cells with both glial and neurocytic differentiation (A - H&E, 40X). In some areas, there were neurocytic rosettes with an eosinophilic neuropil core (B - H&E, 100X). Immunostaining revealed a strong expression of glial fibrillary acidic protein (C - GFAP, 100X), and perhaps most useful, a strong, granular expression of synaptophysin (D - Synaptophysin, 100X).

The patient underwent an endoscopic approach to remove the tumor and complete macroscopic resection was achieved. The tumor was whitish and well adherent to the recesses of the third ventricle. Following the resection of the tumor, a standard endoscopic third ventriculostomy (ETV) was performed. Histopathology showed an RGNT containing two distinct components: a glial component resembling pilocytic astrocytoma, and a neuronal component. Additionally, neurocytic rosettes with an eosinophilic neuropil core were also found (Figure 2).

In the post-operative course, the patient did not develop any neurological deficit and the computed tomography (CT) scan on post-operative day one revealed diminished size of the lateral ventricles, no lesion was observed in the third ventricle and there were no complications related to the surgical procedure. The patient was discharged home in good condition.

After three days of the discharge, the patient presented in the ER with a tonic-clonic seizure and a decreasing level of consciousness. The CT scan did not show hydrocephalus or other complications. On the blood testing of electrolytes, she had hyponatremia with a sodium (Na) level of 119mmol/L. The patient was sent to intensive unit care. Diagnostic tests included urine Na of 87 mmol/L (N<40) and the urinary output was increased (4195ml/24h) with a low urinary density. The cortisol level was low and the thyroid function tests were normal. The patient was accepted to have CSWS with these clinical and laboratory findings. Her hyponatremia resolved completely after two weeks.

**Figure 3:**
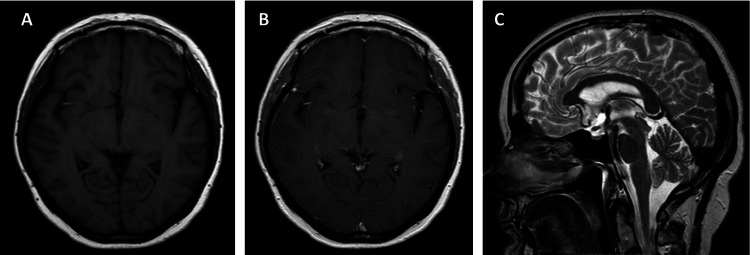
Postoperative MRI - T1 and T1-weighted with no enhancement and no residual tumor. T2 with no recurrence of the tumor and no hydrocephalus.

With two years of follow-up, the patient had no complaints and no tumor progression was observed on postoperative MRIs (Figure 3).

## Discussion

RGNT was first described as a fourth ventricular tumor with the clinical features commonly related to this location like headache secondary to obstructive hydrocephalus and/or ataxia [3]. Different locations of RGNT as chiasma [4], suprasellar region [5], pineal region [6], septum pellucidum [7], intraventricular dissemination [8], spinal cord [9], posterior third ventricle with invasion of thalamus [10] and in the cerebellar hemisphere [11] - outside the midline - have been published lately. To the best of our knowledge, after searching “http://pubmed.gov” with the keyword “rosette-forming glioneural” we only found 10 cases of RGNT involving the third ventricle, and all of them involved only the posterior portion of the third ventricle and invading the posterior or the lateral walls of the third ventricle. We did not find any case involving exclusively the anterior portion of the third ventricle, such as our case illustrated here.

Reviewing only the cases of RGNT of the third ventricle, this type of tumor predominantly affects young subjects with a mean age of 37 years (range: 12 to 79), as in the present case. Contrary to this clinical case and contrary to other locations of RGNT, it shows slight male predominance (M/F ratio: 4) (Table 1). Patients commonly present with headaches, nausea and vomiting, gait instability, and in some cases with visual impairment [12].

**Table 1:** Cases of rosette-forming glioneuronal tumor involving only the third ventricle ETV: endoscopic third ventriculostomy

Author	Title	Age (years)	Gender	Clinical features	Treatment	Follow-up/growth or recurrence
Caled P et al. 2020 [13]	Rosette-Forming Glioneuronal Tumor: An Illustrative Case and a Systematic Review	19	Male	Vomiting, lethargy, gait instability	Craniotomy for resection	36 months/Progression and second lesions
Tanmoy Kumar Maiti et al. 2015 [14]	Rare Pathologies in the Posterior Third Ventricular Region in Children: Case Series and Review	12	Male	Headache, Vomiting, double vision	Craniotomy for total gross resection	9 months/No
H.Cebula et al. 2016 [12]	Thalamic Rosette-Forming a Glioneuronal Tumor in an Elderly Patient: Case Report and Literature Review	75	Female	Headache, drowsiness, gait instability	ETV + Endoscopic biopsy	18 months/No
Ibrahim Alnaami et al. 2013 [10]	Rosette-Forming Glioneuronal Tumors in the Posterior Third Ventricle	57	Male	Headaches, Vomiting, gait instability	ETV + Endoscopic biopsy	6 months/No
Ibrahim Alnaami et al. 2013 [10]	Rosette-Forming Glioneuronal Tumors in the Posterior Third Ventricle	28	Male	Headache, diplopia, mild bilateral lateral gaze palsy	ETV + Endoscopic biopsy	-
Junqing Xu et al. 2012 [15]	Rosette-Forming Glioneuronal Tumor in the Pineal Gland and the Third Ventricle: A Case With Radiological and Clinical Implications	39	Male	Headache, vertigo, memory disorders	Craniotomy for resection	42 months/No
Pankaj Sharma et al. 2011 [5]	Rosette-Forming Glioneuronal Tumors: A Report of Two Cases	16	Female	Headache, diplopia	CT-guided stereotactic biopsy	6 months/No
Pankaj Sharma et al. 2011 [5]	Rosette-Forming Glioneuronal Tumors: A Report of Two Cases	17	Male	Headache	Craniotomy for resection	-
Philip George Eye et al. 2016 [16]	PIK3CA Mutation in a Mixed Dysembryoplastic Neuroepithelial Tumor and Rosette Forming Glioneuronal Tumor, a Case Report and Literature Review	35	Male	Headache, diplopia	ETV + Endoscopic resection	24 months/No
Jian-Qiang Lu et al. 2009 [17]	Multifocal Complex Glioneuronal Tumor in an Elderly Man	79	Male	Gait difficulties	ETV + Endoscopic biopsy	38 months/No
Present case	A Rare Lesion in the Anterior Region of the Third Ventricle: Rosette-Forming Glioneuronal Tumor	44	Female	Headaches, vomiting, decreasing level of consciousness	ETV + Endoscopic resection	24 months/No

All reported cases of RGNT of the third ventricle have been described on the MRI as T1 iso/hypointense and T2 hyperintense [5,10-16]. In this review, six out of ten cases had some different degrees of enhancement, probably related to soli areas. The remaining four cases did not show contrast enhancement [5,13,16]. In the present case, the lesion showed a mild dotted enhancement inside the mass with a hypointense signal in the gradient-echo (T2*) which could represent calcification or small intralesional bleeding. These features were only observed in one case of a left posterior thalamic lesion [12].

Total or subtotal resection by craniotomy was achieved in four cases [5,13-15] and five biopsies were performed: one of them was a CT-guided stereotactic biopsy [5] and four of them were an endoscopic tumor biopsy [10,12,17].

Only in one case, the patient underwent endoscopic resection of the third ventricular component of the tumor but no information was provided about the size of the resection [16]. In all cases of endoscopic approach, a third ventriculostomy was performed [10,12,16,17]. Adjuvant radiotherapy was employed only in the case that the patient was submitted to a CT-guided stereotactic biopsy [5]. According to our research, this is the first case of purely neuroendoscopic management and total resection of an RGNT exclusively of the anterior portion of the third ventricle.

In the present case, our patient developed a rare clinical condition with tonic-clonic seizures probably due to a CSWS. According to our research, there is no similar case reported of this clinical condition in patients with RGNT of the third ventricle who underwent endoscopic resection or biopsy. Neuroendoscopy is recognized as a safe technique, but the risk of complications still exists, especially because orientation, navigation, and direct visualization may be not straightforward with abnormal anatomy. Reported complication rates vary from 6% to 14% and most frequently they are associated with ETV [18]. The floor of the third ventricle is thinned out and essentially transparent and the hypothalamic nuclei are displaced laterally. Injury to the hypothalamic-pituitary axis with associated endocrinological and electrolyte abnormalities following ETV has been rarely reported in the literature [18]. Diabetes insipidus and hypernatremia have been more commonly reported than hyponatremia. The pathogenesis may be related to supraopticohypophyseal tract functional disruption or damage to the subforniceal organ during surgery [19].

There has been limited literature specifically discussing hyponatremia after ETV and when it occurs, it is more often associated with the syndrome of inappropriate secretion of antidiuretic hormone (SIADH) than CSWS [18,20].

In a case reported by Vaicys and Fried, they presented an infant, with an 11-month-old male, who underwent ETV due to symptomatic hydrocephalus secondary to aqueductal stenosis and after four days he developed absence seizures due to hyponatremia (Na of 117mEq/l) with a SIADH. Hyponatremia was corrected up to 139mEq/l by fluid restrictions within 24 hours [18].

Shih-Shan Lang et al. reported 32 patients who underwent ETV for management of hydrocephalus and five patients (16%) developed symptomatic hyponatremia between one and eight days following the procedure. In two patients, the initial manifestation of hyponatremia was a seizure. Four of five patients with hyponatremia were not in the hospital when the signs of hyponatremia initially manifested, so they were unable to ascertain the volume status of the patients, therefore they were incapable of proving definitively whether hyponatremia was caused by SIADH or CSWS, but the hyponatremia was treated in all of the patients with a combination of fluid restriction and sodium supplementation which go in favor of SIADH [19].

Isik and Ozek reported a male infant diagnosed in utero with a suprasellar arachnoid cyst and treated with endoscopic ventriculocystocisternotomy on the 38th day of life and after that, he developed hyponatremia with urine NA of 71mmol/L and increased urinary volume (3.4ml/kg(hour)). The patient received fluids and his hyponatremia resolved completely one month after the surgery. The patient was accepted to have CSWS with these clinical and laboratory findings [20]. This was the first clinical case reported a CSWS after ETV on a child. To the best of our knowledge, our present case is the first case of a well-reported CSWS after ETV, associated with seizures in an adult.

In our case, the intimate relation of the tumor with the recesses of the third ventricle and the cold and/or rapid irrigation into the ventricular system could be responsible for the development of hyponatremia [19,20].

With 24 months of follow-up, our patient had no complaints, the MRI demonstrated a functioning ETV and no tumor recurrence or progression was observed. The cases previously published of RGNT in the third ventricle had an average follow-up of 22.38 months (range: 6-38 months). Only one case showed a significant tumor progression and the appearance of a second lesion and this patient had been submitted to a craniotomy for resection of the tumor [13]. There are a few cases of late recurrence despite gross total resection and some cases of malignant transformation of RGNT after a total or subtotal resection. On the other hand, Zhan et al. [1] reported that selected cases of RGNT could be managed conservatively, considering, the slow-growing behavior of such a tumor. We need more cases and a long-term follow-up to understand the biological behavior and clinical outcome of RGNT.

## Conclusions

In this article, we present a unique case of an RGNT localized exclusively in the anterior portion of the third ventricle and we claim that minimally invasive techniques such as neuroendoscopy may enable adequate diagnosis and management of symptoms in selected patients, never forgetting the inherent risks of this surgical technique even the rarest.
